# Validation of livability environmental limits to heat and humidity

**DOI:** 10.1152/japplphysiol.00225.2024

**Published:** 2024-11-14

**Authors:** Xiaojiang Xu, Timothy P. Rioux, John W. Castellani, Scott J. Montain, Nisha Charkoudian

**Affiliations:** ^1^Thermal and Mountain Medicine Division, U.S. Army Research Institute of Environmental Medicine, Natick, Massachusetts, United States; ^2^Montana Center for Work Physiology and Exercise Metabolism, College of Health, The University of Montana, Missoula, Montana, United States

**Keywords:** climate change, global warming, heat stress, livability, thermoregulatory model

## Abstract

Rising global temperatures, driven by climate change, pose a threat to human health and regional livability. Empirical data and biophysical model-derived estimates suggest that the critical environmental limits (CELs) for livability are dependent on ambient temperature and humidity. We use a well-validated, physiology-based, six-cylinder thermoregulatory model (SCTM) to independently derive CELs during sustained minimal, light, and moderate activity across a broad range of ambient temperatures and humidity levels and compare with published data. The activity and environments were considered livable if predicted core temperatures did not reach 38 ± 0.25°C within 6 h. The outcomes for minimal activity revealed CELs ranging from 34°C/95% relative humidity (RH) to 50°C/5% RH. Corresponding dry heat losses ranged from 14 to −72 W·m^−2^ (negative = heat gain) and evaporative heat losses ranged from 39 to 104 W·m^−2^. The wet-bulb temperature (*T*_wb_) at the CELs ranged from 33.3°C to 20.9°C. Activity shifted CELs toward lower temperatures and humidities. Importantly, our predicted CELs largely agree with observed livability CELs from physiology and those from a biophysical model. The physiology-grounded SCTM has utility for assessing the impact of climate change on regional livability.

**NEW & NOTEWORTHY** This study is the first to use a physiology-grounded thermoregulatory model to predict critical environmental limits (CELs) above which human thermoregulatory capacity is exceeded. The model outcomes closely approximate empirically derived CELs, showing it is a strong model for estimating and preparing for the impact of climate warming on local, regional, and world human population livability and migration.

## Graphical abstract

**Figure:**
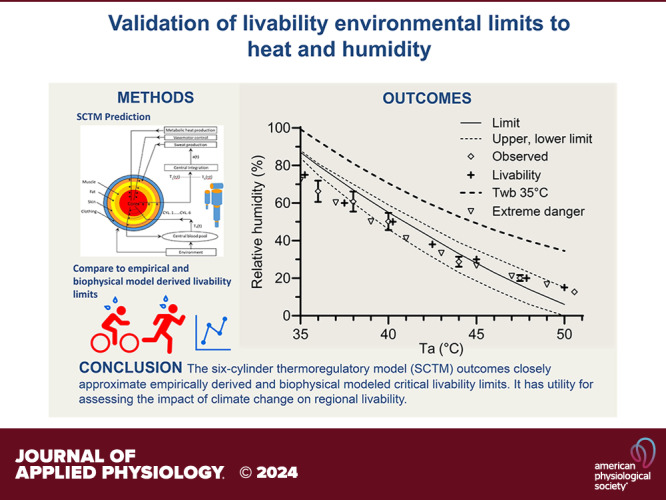

## INTRODUCTION

Climate change is causing global air temperatures to progressively increase worldwide. July 2023 was one of the hottest months ever recorded in the Northern Hemisphere, with southern Europe recording a new maximum daily temperature record of near 50°C and some countries in Asia reporting high temperatures above 50°C. In the United States, daily highs in Phoenix, Arizona, reached approximately 43°C for 31 consecutive days and the highest temperature in Death Valley National Park in California was 53.3°C (1). These extreme environmental temperatures prompt essential questions, such as, what are the upper environmental conditions for human livability during minimal activity and how do these limits shift during light and moderate activity?

In 2010, a wet-bulb temperature (*T*_wb_) of 35°C was proposed as the adaptability limit for humans under sustained environmental heat stress, but was based on a simple analysis of basic heat transfer and physiological principles (2) and predicted survivability rather than livability. In 2022, human physiological studies (PSU H.E.A.T. project) using six different graded progressive heat stress tests during sustained minimal activity were executed to establish the critical environmental limits (CELs), above which body heat transfer was compromised and progressive increases in body core temperature occurred (3, 4). These studies revealed that the livability CELs did not occur at a fixed *T*_wb_ and instead ranged from an approximate *T*_wb_ of 26–31°C, with lower values observed in hot-dry environments and higher values in warm-humid conditions. Vanos et al. (5) subsequently used partitional calorimetry to estimate the upper heat stress conditions, which could be tolerated before progressive heat storage would occur during minimal and several levels of physical activity. Their resulting livability curves (similar to CELs) revealed that *T*_wb_ at the minimal activity threshold fell from about 32°C in warm air temperatures to 22°C in hot air temperatures, and the same pattern persisted at higher activity levels, confirming that no single *T*_wb_ is sufficient as a livability index. The biophysical model used by Vanos et al. (5), however, included several assumptions, including that mean skin temperatures and sweat rates were constant and the same values were used across all the conditions studied. Based on the PSU H.E.A.T. data, a conceptual model for human heat tolerance has been proposed (6). However, this model is also a steady-state model and does not include the dynamics of the thermoregulatory system during exposures.

Human thermoregulatory models have proven to be effective and efficient tools for simulating and analyzing the interactive relationships among environmental conditions, clothing, and metabolic heat production (7–10). These models can predict various parameters (e.g., survival time, skin temperatures, core temperatures) and are important tools in multiple fields of study including search and rescue, medicine, public health, and physiology. The well-validated six-cylinder thermoregulatory model (SCTM) is one such model (11–15). It is a rational model based on the first principles of heat transfer and physiological control mechanisms. The human body is subdivided into six segments representing the head, trunk, arms, legs, hands, and feet. Each segment is further divided into concentric compartments representing the core, muscle, fat, and skin. The SCTM simulates the integration of afferent thermal signals and activation of the thermoregulatory effectors of shivering heat production, vasodilation/vasoconstriction, and sweat production. The model inputs include individual characteristics, environmental conditions, metabolic activity, and clothing parameters. The SCTM predicts core temperatures, skin temperatures, and sweat rates for each of the six body regions.

The purpose of this article is to apply the SCTM to simulate human thermoregulatory responses during minimal but sustained activities under a wide range of environmental conditions to determine if the model-derived CELs acceptably agree with the experimentally and biophysical model-derived values (3, 4) and to examine how light to moderate activity levels affect the CELs. Finally, we compare the predicted CELs during the minimal activity level to the heat index levels deemed extremely dangerous by the US National Oceanic and Atmospheric Administration (16).

## MATERIALS AND METHODS

### Physiological Thresholds

Body core temperature is the primary physiological parameter used to assess the health risks of heat stress. For our CEL determinations, we chose 38 ± 0.25°C as the critical upper limit for body core temperature. Our rationale for selecting this temperature criterion came from the World Health Organization’s (WHO) scientific group on heat stress who have suggested 38°C as an upper limit deep body temperature for prolonged daily exposures to heavy work (17). This is also the acceptable core temperature during heat exposure that is used by the International Organization for Standardization (ISO) and the National Institute for Occupational Safety and Health (NIOSH) (18–21). Analysis of physiological data further showed that the core temperature of 38°C is a reasonable threshold for sustainable heat exposure (22). Furthermore, the 38°C threshold was well above the predicted steady state during minimal (37.2°C), light (37.4°C), and moderate work (37.6°C) performed in the climatic conditions within the thermal neutral zone (23, 24). To accommodate individual differences in this threshold (22), deep body temperatures of 37.75 and 38.25°C were included in the validation as lower and upper thresholds for sustainable heat exposure.

### Critical Environmental Limits

To derive SCTM CELs, individual simulations at ambient temperatures set at 28–50°C in increments of 2°C, with relative humidity (RH) ranging from 5 to 95% in increments of 5%, and wind speed, 0.2 m·s^−1^ were conducted. The solar load was excluded from the simulations so the outcomes could be directly compared with the empirically derived CELs in the literature. The simulation duration was set at 6 h. This length was purposefully conservative to ensure sufficient run times for steady-state core temperatures to be achieved (if they were going to occur). It was assumed that if core temperature had not reached 38°C within the 6-h time limit, it was unlikely that the activity and climatic load would produce core temperatures above the threshold unless some other condition, such as dehydration, led to a further rise in body core temperatures. The simulations assumed a T-shirt, light pants, and shoes (summer clothing) were worn. The intrinsic thermal and evaporative resistances for this ensemble were 0.065 m^2^°C·W^−1^ and 0.0088 m^2^·kPa·W^−1^, respectively. The human physical characteristics were set to a height of 1.7 m, weight of 78 kg, and 19% of body fat. These were deemed acceptable as they approximate the size and body composition of an average young man. The simulations were performed at three metabolic rates, 155 W (0.464 L·min^−1^ V̇o_2_), 250 W, and 350 W. The metabolic rate of 155 W was used to reflect the metabolic demand of minimal physical activities of daily living and in comparison to the literature CELs (3, 25). Activity at metabolic rates of 250 and 350 W was included in the experiment to examine the effects of metabolic rates associated with occupational labor on the CELs for any given environmental condition.

The SCTM used the above inputs to predict human thermoregulatory responses across the different combinations of ambient temperature, relative humidity, and metabolic rate with the results used to determine critical environmental limits. The activity level in any environmental condition was considered sustainable if the predicted core temperatures remained below 38°C during the 6-h simulation period. The environmental conditions were considered too oppressive for sustainable activity if the predicted core temperatures reached 38°C or above during the 6-h simulation period. At ambient temperatures where the 38°C threshold was exceeded, the CEL was set at the lowest humidity when the threshold was reached. Therefore, our CEL is the environmental condition(s) when the core temperature reached 38°C within 6 h while the livability CEL in the PSU H.E.A.T. studies and in the biophysical modeling effort were the environmental conditions that body heat balance could not be maintained (3–5).

### Six-Cylinder Thermoregulatory Model

As previously stated, the SCTM is a rational thermoregulatory model and is based on the first principles of physiology and the physical laws of heat transfer (11, 12). The SCTM simulates human temperature regulation, controlled by central (mostly hypothalamic) sites, which integrate thermal inputs from both central and peripheral thermoreceptors to elicit appropriate efferent thermoregulatory responses (26). When afferent information indicates increases in body temperature, reflex heat dissipation effectors (sweating, cutaneous vasodilation) are activated to counteract these increases in a negative feedback loop (27, 28). When afferent information indicates decreases in body temperature, reflex heat conservation and/or generation effectors (cutaneous vasoconstriction, shivering, non-shivering thermogenesis) engage to counteract these decreases. For the present SCTM simulations, the initial deep body temperature was 36.83°C. Details of the SCTM, e.g., equations, numerical method, and initial validation, can be found in previous papers (11, 12), and follow-on evaluations across a broad range of conditions, e.g., high-intensity exercise and water immersion, can be found in additional papers (13–15). The equations for body heat losses are briefly described below.

The human body exchanges heat with the environment through its skin surface by radiation, convection, and evaporation. Convection, radiation, and evaporation all contribute to the heat exchange at the boundary and the boundary condition is described as:

(*1*) $$-\lambda \frac{\partial T}{\partial r}=R+C+E$$

where λ is the thermal conductivity of the tissue (W·m^−1^·°C^−1^), *T* is the tissue temperature (°C), *r* is the radius (m), *R* is the radiative heat exchange (W·m^−2^), *C* is the convective heat exchange (W·m^−2^), and *E* is the evaporative heat exchange (W·m^−2^). The *E* is predicted by SCTM, primarily determined by the efferent signal, and restricted by skin, clothing, and environmental conditions.

Assuming the mean radiant temperature is equal to the ambient temperature (*T_a_*), the dry and maximal evaporative heat losses from the body surface to the environment are described by

(*2*) $$R+C=\frac{T_{s}-T_{a}}{R_{cl}+\frac{1}{f_{cl}\cdot (h_{c}+h_{r})}}\;[\text{W}\cdot {\text{m}}^{-2}]$$

(*3*) $$E_{\text{max}}=\frac{P_{sk,s}-P_{a}}{R_{cl,e}+\frac{1}{f_{cl}\cdot h_{c}\cdot LR}}\;[\text{W}\cdot {\text{m}}^{-2}]$$

where *T_s_* is the skin temperature (°C); *R_cl_* is clothing intrinsic resistance (m^2^·°C·W^−1^); *f_cl_* is clothing area factor, the ratio of the clothing surface to body surface area (dimensionless); *h_c_* is convective heat transfer coefficient (W·°C^−1^·m^−2^); *h_r_* is radiative heat transfer coefficient (W·°C^−1^·m^−2^); *E*_max_ is the maximal evaporative heat loss (W·m^−2^); *P_sk,s_* is the saturated water-vapor pressure at the skin (kPa); *P_a_* is water-vapor pressure of the ambient environment (kPa); *R_cl,e_* is clothing intrinsic evaporative resistance (m^2^·kPa·W^−1^); *LR* is the Lewis ratio, 0.0165°C·Pa^−1^. The *E*_max_ reduces as the vapor pressure *P_a_* of the environment increases and *E*_max_ reduces to zero in warm and high-humidity environments when the saturated vapor pressure at the skin is equal to the vapor pressure of the environments.

SCTM inputs include individual characteristics (i.e., height, weight, and body fat percentage), environmental (i.e., air/water temperature, humidity, and wind velocity), and clothing (clothing insulation, moisture permeability index) parameters for each of the six cylinders. Outputs from the model include predicted physiological responses in each of the six body regions, including core/deep body temperature (the center of the torso cylinder), skin temperatures, and sweating rates.

### Wet-Bulb Temperature

The *T*_wb_ is the adiabatic saturation temperature and is measured by a thermometer with a bulb, which is covered by a wet cloth. At low wind speeds, *T*_wb_ can be estimated from the temperature and relative humidity with the following equation (29):

(*4*) $$\begin{array}{l} \begin{array}{l} T_{\text{wb}}=T_{a}\cdot \text{atan}[0.151977\cdot {\left(\text{RH}\%+8.313659\right)}^{\frac{1}{2}} \end{array}\\ \;\;+\;\text{atan}\left(T_{a}+\text{RH}\%\right)-\text{atan}\left(\text{RH}\%-1.676331\right)\\ \;\;+\;0.00391838\cdot \text{RH}{\%}^{\frac{3}{2}}\cdot \text{atan}\left(0.023101\cdot \text{RH}\%\right)\\ \;\;-\;4.686035 \end{array}$$

## RESULTS

**Figure 1.**
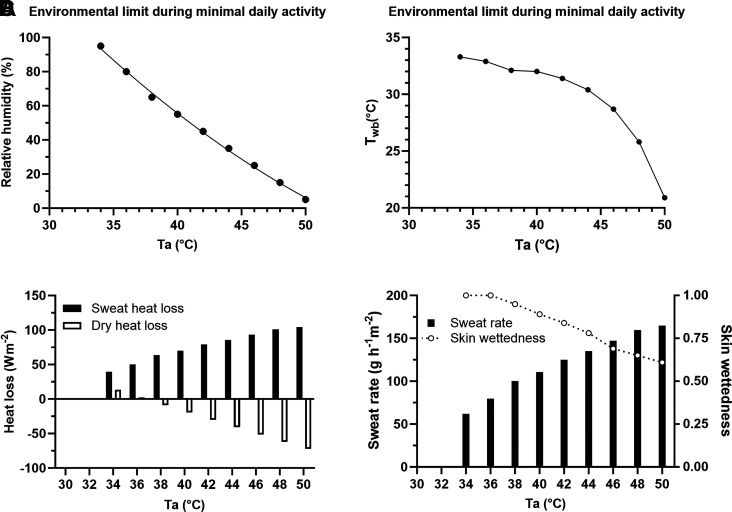
Critical environmental limits during minimal daily activity at a metabolic rate of 155 W (*A*, *top left*), RH = 0.0852·*T*_a_^2^12.617·*T*_a_ + 423.98 (*R*^2^ = 0.998). Critical environmental limits in *T*_wb_ (*B*, *top right*), sweat evaporative heat loss and dry heat loss (*C*, *bottom left*), and sweat rate and skin wettedness (*D*, *bottom right*). Negative values for dry heat loss indicate heat gain from environments. RH, relative humidity.

Figure 1 presents the simulation outcomes during minimal daily activity (metabolic rate 155 W) across the range of ambient temperatures and humidity conditions that were examined. In Fig. 1*A*, the environmental conditions below or to the left of the CEL line are sustainable (deep body temperature is preserved below 38°C over a period of 6 h) while those above or to the right of the line are unsustainable (>38°C). When the environmental temperatures were 50°C or above, the environmental conditions were unsustainable regardless of the relative humidity. The CEL curve is a second-order polynomial regression curve that expresses the CEL, critical relative humidity, as a function of ambient temperature, 34°C ≤ *T_a_* ≤ 50°C. Figure 1*B* displays the *T*_wb_ corresponding to the predicted CELs and ranged from 33.3°C in the warm but humid conditions to 20.9°C in the hottest but dry conditions. Figure 1*C* presents the dry heat and evaporative heat losses corresponding to the CELs. As the environmental temperature increased, dry heat loss became increasingly negative, and the human body gained heat from the environment through convection and radiation. Under these conditions, sweat evaporation was the sole mechanism to dissipate heat. Dry heat losses in these simulations ranged from 13 W to –72 W·m^−2^. Correspondingly, evaporative heat losses ranged from 39 to 104 W·m^−2^ (∼62–165 g·h^−1^·m^−2^). Figure 1*D* presents the impact of the CEL environmental conditions on the model-derived sweating rate and skin wettedness.

**Figure 2.**
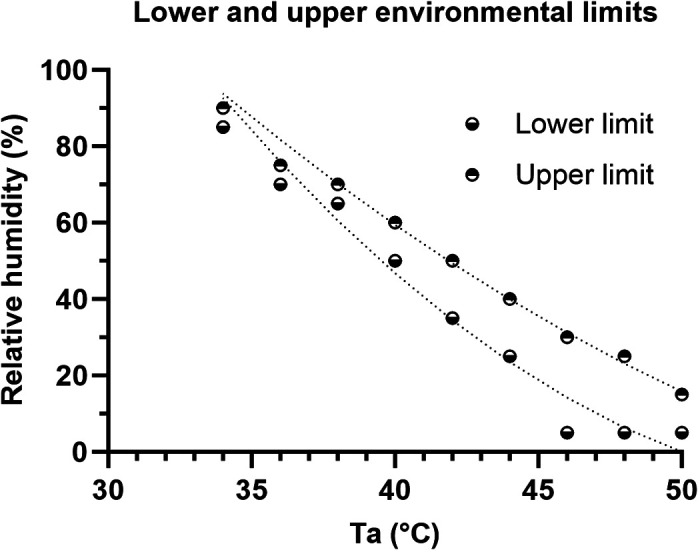
Upper and lower critical environment limits during minimal daily activily at a metabolic rate of 155 W. The upper limits indicate that the core temperature increased to 38.25°C, RH = 0.1853·*T*_a_^2^ − 21.367·*T*_a_ + 604.7 (*R*^2^ = 0.997) and lower limits indicate the core temperature increased to 37.75°C, RH = 0.0805·*T*_a_^2^ − 12.03·*T*_a_ + 404.5 (*R*^2^ = 0.998). RH, relative humidity.

Figure 2 illustrates the predicted threshold environmental conditions that reached the lower and upper threshold body core temperatures of 37.75°C and 38.25°C, respectively, during minimal activity level simulation. The conditions between the low and upper limits can be considered the transition area where the heat stress is transitioning from sustainable (under the lower limit) to unsustainable (above the upper limit). The curves were second-order polynomial regression curves that expressed the upper CEL and lower CEL as a function of ambient temperature, 34°C ≤ *T_a_* ≤ 50°C.

**Figure 3.**
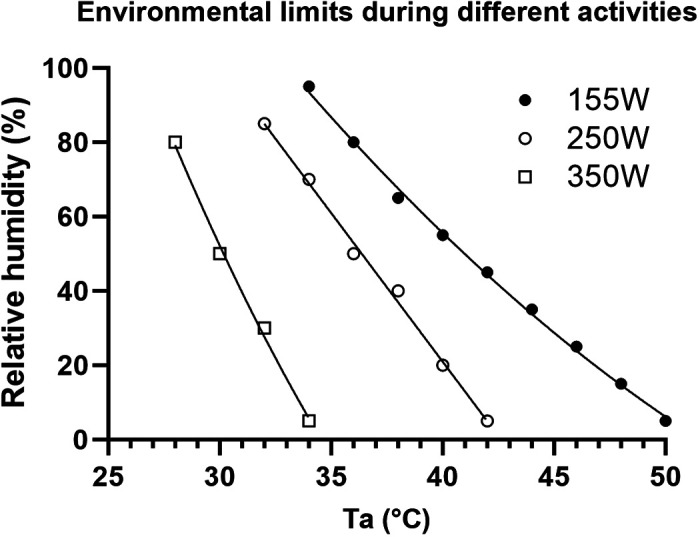
Critical environmental limits during exercise at metabolic rates of 155, 250, and 350 W.

Figure 3 illustrates how metabolic rate impacts the CELs. For each metabolic rate, the environmental conditions below and to the left of the limit line are sustainable using the criterion that body core temperature should not exceed 38°C over 6 h of sustained effort. Environmental conditions above and to the right resulted in body core temperatures in excess of 38°C within 6 h of simulated activity. As illustrated in Fig. 3, there was an incremental leftward and downward shift in the CEL at metabolic rates of 250 and 350 W.

**Figure 4.**
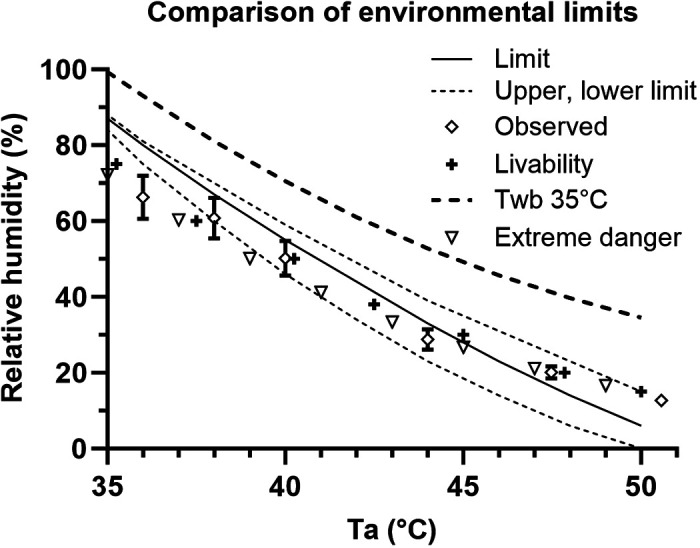
Comparison of our predicted environmental limits (— SCTM), with lower and upper limits during minimal daily activity (- - - SCTM), with observed upper environmental limits for body core temperature stability (◊ PSU H.E.A.T. data) (3, 4), the theoretical limit of *T*_wb_ 35°C (-- -- - Sherwood et al., 2), the calculated limits of livability during minimal activity (**+** Vanos et al., 5), and conditions when the extreme danger limit of the Heat Index have been reached (**∇** NWS) (30). SCTM, six-cylinder thermoregulatory model.

To examine how our predicted outcomes compare with the existing literature, we combined our predicted CELs for the 155 W workload and the associated upper and lower boundary CELs with the proposed *T*_wb_ 35°C curve, the CELs derived from human experimentation (3, 4), the estimated limits of livability for light activity (5) and extreme danger limit of Heat Index used by US National Weather Service (16, 30). The latter was determined using the NWS Heat Index Calculator (30) and refers to conditions when heat stroke risk reaches a probability of being highly likely (16). The combined data are presented in Fig. 4.

## DISCUSSION

The outcomes of this study provide validation to the CELs derived in the PSU H.E.A.T. studies using a ramped heat stress protocol during minimal activity and to the livability limits presented by Vanos et al. using biophysical modeling (3–5). The agreement among these CELs despite three different experimental approaches is a testament to the robustness of these CELs as measures for livability analyses. Our data also share the magnitude of leftward and downward shift to the CELs when light and moderate sustained work is attempted in the same climatic heat stress conditions and the attainment of a body core temperature of 38°C is used as the upper limit. Together, these outcomes provide support for the use of the SCTM for examining physical conditions, such as clothing, or individual factors, such as body size and fat percentage, on the CELs and toward the potential derivation of new indices of livability.

To our knowledge, the present report is the first attempt to use a physiology-based thermoregulatory model to derive estimates for the critical environmental limits for sustained body temperature regulation. By using the SCTM, we were able to robustly examine the interaction of ambient temperature and humidity on human thermoregulatory responses at metabolic rates approximating sedentary activities as well as light and moderate levels of sustained activity over a 6-h period. Consistent with previous physiological studies attempting to define these limits (3, 4, 31), we found that the CEL at any given temperature was dependent on the humidity. For the CEL at 35°C, the relative humidity would need to be in excess of 80% but at air temperatures in excess of 40°C, the CEL was reached at much lower humidity levels. Furthermore, light and moderate physical activity incrementally shifted the CELs leftward and downward; in other words, the CELs occurred at a lower ambient temperature and humidity combinations with the magnitude of the shift dependent on the intensity of the activity. Finally, a single *T*_wb_ was insufficient to define a CEL setpoint.

Our predicted CELs during minimal activity are generally in agreement with the PSU H.E.A.T empirically observed critical limits (3, 4), particularly above 37°C. Across the range of ambient temperatures examined, the differences between modeled and empirically derived CELs ranged from −7.7 to 13.8% RH with a standard deviation of 7.7% RH. Moreover, the empirically derived CELs fell within the SCTM-predicted lower and upper limits in five of six available comparisons. For the PSU H.E.A.T. data, young human volunteers were exposed to progressive environments and performed tasks at minimal metabolic rate mimicking basic activities of daily life (3, 25). The environmental conditions were stable for 30 min, then the vapor pressure or the ambient temperature was increased by 1 mmHg or 1°C every 5 min until a clear rise in the core temperature was observed. Although our method and the PSU H.E.A.T. method in determining CELs are different, the similarity of the predicted limits offers support for accepting the validity of the CEL estimates. Our CELs are also in agreement with the limits of livability derived by Vanos et al. (5) for minimal activity, when air temperatures are above 40°C. Likewise, our minimal activity CELs were in agreement with the Heat Index (16), which is widely used by the US National Weather Service for heat stroke risk warnings when ambient temperatures are 40°C and higher. In contrast, the SCTM produced CELs that were higher than the empirical and biophysical-derived CELs of PSU H.E.A.T (3, 4) and Vanos et al. (5), respectively, in 35–37°C ambient temperature range. We suspect the SCTM model’s modestly higher CELs in this ambient temperature range are in part due to the SCTM assuming 100% sweat efficiency when skin wettedness is 0.8 and higher. It is known that sweat efficiency falls when skin wettedness reaches 0.8 and higher, and this loss of efficiency would have led to greater heat gain than the model predicted. The higher CELs might also be due to our choice to use the attainment of 38°C body core temperature as the CEL threshold value. Inspection of the time series data reveals that body core temperatures were progressively rising at the humidity levels consistent with the empirically derived CEL conditions, but did not rise enough to reach the 38°C threshold within the 6 h period.

Evaporative heat loss (latent heat loss) is a physiological and physical phenomenon while dry heat loss (sensible heat loss) is a physical phenomenon solely dependent on temperature gradients between skin and environments. Evaporative heat loss is dependent on the amount of sweat secreted on the skin (physiological thermoregulation) and the physical conditions on and near the skin that determine if the sweat evaporates and is converted into heat loss. In Fig. 1*C*, the predicted dry losses from 36°C to 50°C ranged from 2 to −72 W·m^−2^, and evaporative heat loss ranged from 50 to 104 W·m^−2^. These values are compatible with the observed values, about 2 to −77 W·m^−2^ for dry heat loss and 61 to 107 W·m^−2^ for evaporative heat loss (4). When the environmental temperature becomes higher than the skin temperature, the dry heat loss decreases and ultimately becomes a heat gain around *T_a_* 36°C to 38°C (i.e., the body gains heat from the environments). Under these conditions, sweat evaporation becomes the sole mechanism for heat loss and is the key for body heat balance. As shown in Fig. 1, the evaporative heat loss was 50 W·m^−2^ (79 g·h^−1^·m^−2^) at 36°C/80% RH and reached 104 W·m^−2^ (165 g·h^−1^·m^−2^) at 50°C. Usually, only a portion of sweat secreted evaporates for various reasons, for example, sweat dripping off the skin, restricting maximal evaporative capacity (32–35). If the sweat efficiency is assumed to be 0.75, then sustainable sweat rate would be at least 105 g·h^−1^·m^−2^ or higher to live at this estimated limit of hot and humid environments. Thus, CELs are directly dependent on the sustainable sweat rates of the human being.

A new addition to the expanding CEL knowledge base is the pronounced shift in the CELs accompanying the light and moderate physical activity levels relative to the CELs for minimal activity. The SCTM predicted that at 250 W, there would be a 3–7°C leftward ambient temperature shift in the CEL threshold and this level of activity would not be sustainable (i.e., keep body core temperature below 38°C) for 6 h in temperatures above 41°C. Likewise, the 350 W activity level shifted the CEL ambient temperature threshold 7–14°C leftward from the minimal activity CEL at the same humidity level, and moderate activity level was not predicted to be sustainable above 33°C ambient temperature, independent of humidity.

These predicted CELs have value for assessing the impact of climate change on human health and livability but have limitations. We suspect that the 38°C threshold was too conservative for deriving the moderate activity level CELs, as several of the core temperature profiles at this activity level reached steady state above the 38°C threshold. If we had applied the same progressive heat storage criteria as Vanos et al. (5), our CELs would have been essentially identical to their 350 W livability limits. Separately, health status, older age, lack of acclimatization, and individual fitness levels, etc. (36–41) will likely create individual variability and could lessen the ability to dissipate the heat load and lower or leftward shift the CELs. For example, elderly adults have impairments of thermoregulatory functions with attenuated physiological ability to increase skin blood flow and generate sweat (37, 39, 42), recent data demonstrated that CELs for older adults are relatively lower than for young adults (43) and certain individuals have compromised sweating, such as occurs with diabetic and other peripheral neuropathies (44), and thus are more vulnerable to morbidity and mortality in heat. The heat burden of solar radiation was also not considered in the assessment. No simulations or experiments performed to date have examined the influence of added solar load. In addition, the SCTM assumes that over the 6-h simulation period, the human system functions normally, without any heat-related issues, such as impaired thermoregulation, heat exhaustion, cardiovascular stress, and kidney stress. Thus, the predicted CELs might very well be too liberal for certain populations and environmental conditions.

### Conclusions

The SCTM, a rational thermoregulatory model, successfully predicted CELs that were largely in agreement with empirically derived livability CELs and those derived separately using a biophysical model. Therefore, the SCTM is a strong model for estimating and preparing for the impact of climate warming on local, regional, and world human population livability and migration. Consistent with others, we found that the minimal activity CELs did not occur at a single *T*_wb_, decreasing from 33 to 21°C as ambient temperatures increased from 34°C to 50°C, respectively. Light activity shifted the CELs toward lower temperatures and humidities in a graded manner. This physiology-grounded mathematical model has utility for assessing the impact of climate change on livability in the heat.

## DATA AVAILABILITY

Data will be made available upon reasonable request.

## DISCLAIMERS

The opinions or assertions contained herein are the private views of the authors and are not to be construed as official or as reflecting the views of the Army, or the Department of Defense. Any citations of commercial organizations and trade names in this report do not constitute an official Department of the Army endorsement or approval of the products or services of these organizations.

## DISCLOSURES

No conflicts of interest, financial or otherwise, are declared by the authors.

## AUTHOR CONTRIBUTIONS

X.X. conceived and designed research; X.X., T.P.R., and S.J.M. analyzed data; X.X., J.W.C., S.J.M., and N.C. interpreted results of experiments; X.X. prepared figures; X.X., J.W.C., and S.J.M. drafted manuscript; X.X., T.P.R., J.W.C., S.J.M., and N.C. edited and revised manuscript; X.X., T.P.R., J.W.C., S.J.M., and N.C. approved final version of manuscript.
